# Supplementary material for: Denuded peptidoglycan oligosaccharides enable the biochemical investigation of bacterial cell wall recognition, modification, and degradation
Source: J Biol Chem. 2026 Aug 17;302(10):113462. doi: 10.1016/j.jbc.2026.113462 (PMC13627109; doi:10.1016/j.jbc.2026.113462)
Supplement: Supplementary file 2 [file mmc2.docx]

| **Table S1. List of PG cleaving enzymes and their known catalytic activities used in this study** | | | |
| --- | --- | --- | --- |
|  | **Full Enzyme Name** | **Known Catalytic Activity** | **Uniprot Accession Number** |
| **MltA** | Membrane-bound lytic murein transglycosylase A | Exolytically cleaves the 1-4 β-glycosidic linkage between NAM and NAG, forming a 1,6-anhydro-NAM product. | Q9I4B6 |
| **MltB** | Soluble and membrane-bound lytic transglycosylase | Cleaves the 1-4 β-glycosidic linkage between NAM and NAG, forming a 1,6-anhydro-NAM product. | Q9HVX3 |
| **MltD** | Membrane-bound lytic murein transglycosylase D | Cleaves the 1-4 β-glycosidic linkage between NAM and NAG, forming a 1,6-anhydro-NAM product. | Q9I2T2 |
| **MltG** | Endolytic murein transglycosylase | Cleaves the 1-4 β-glycosidic linkage between NAM and NAG, forming a 1,6-anhydro-NAM product. | Q9HZN7 |
| **MltF** | Membrane-bound lytic murein transglycosylase F | Exolytically cleaves the 1-4 β-glycosidic linkage between NAM and NAG, forming a 1,6-anhydro-NAM product. | Q9HXN1 |
| **MltF2** | Glycosylase | Cleaves the 1-4 β-glycosidic linkage between NAM and NAG, forming a 1,6-anhydro-NAM product. | Q9HZY1 |
| **Slt70** | Soluble lytic transglycosylase | Cleaves the 1-4 β-glycosidic linkage between NAM and NAG, forming a 1,6-anhydro-NAM product. | Q9HZI6 |
| **SltB1** | Soluble lytic transglycosylase B | Cleaves the 1-4 β-glycosidic linkage between NAM and NAG, forming a 1,6-anhydro-NAM product. | Q9HX24 |
| **SltB2** | Transglycosylase | Cleaves the 1-4 β-glycosidic linkage between NAM and NAG, forming a 1,6-anhydro-NAM product. | Q9I4G6 |
| **SltB3** | Lytic murein transglycosylase | Cleaves the 1-4 β-glycosidic linkage between NAM and NAG, forming a 1,6-anhydro-NAM product. | Q9HX28 |
| **RlpA** | Endolytic peptidoglycan transglycosylase RlpA | Cleaves the 1-4 β-glycosidic linkage between NAM and NAG, forming a 1,6-anhydro-NAM product. Strong preference for glycans lacking stem peptides. | Q9X6V6 |
| **NamZ** | Peptidoglycan beta-N-acetylmuramidase NamZ | Exolytically cleaves the non-reducing NAM of peptidoglycan chains | P40407 |
| **NagZ** | Beta-hexosaminidase | Exolytically cleaves the non-reducing NAG of peptidoglycan chains | P40406 |

| **Table S2. Enzymes and plasmids used in the study** | | | | | |
| --- | --- | --- | --- | --- | --- |
| Gene | Source | Plasmid name | Plasmid backbone | Details | Expression strain |
| *MltA* (PA1222) | *Pseudomonas aeruginosa* PAO1 | pACCV-4 | pET28a(+) | truncated aa 1-25 | E. coli BL21(DE3) Star |
| *MltB* (PA4444) |  | pNBAC54-1 | pET30a(+) | Ref: <https://pubs.acs.org/doi/10.1021/bi011833k> | E. coli BL21(DE3) pLysS |
| *MltD* (PA1812) |  | pACCV-32 | pET28a(+) | truncated aa 1-34 | E. coli BL21(DE3) Star |
| *MltF* (PA3764) |  | pACCV-21 | pET28a(+) | truncated aa 1-38 | E. coli SHuffle T7 |
| *MltF2* (PA2865) |  | pACCV-31 | pET28a(+) | truncated aa 1-21 | E. coli BL21(DE3) Star |
| *MltG* (PA2963) |  | pACCV-37 | pBAD-SUMO | truncated aa 1-24 | E. coli BL21(DE3) Star |
| *RlpA* (PA4000) |  | pACCV-33 | pET28a(+) | truncated aa 1-29 | E. coli BL21(DE3) Star |
| *Slt70* (PA3020) |  | pACCV-47 | pET28a(+) | truncated aa 1-25 | E. coli BL21(DE3) Star |
| *SltB1* (PA4001) |  | pNBAC258-2 | pET30a(+) | Ref: <https://pubs.acs.org/doi/10.1021/bi011833k> | E. coli BL21(DE3) pLysS |
| *SltB2* (PA1171) |  | pACCV-29 | pET28a(+) | truncated aa 1-18 | E. coli BL21(DE3) Star |
| *SltB3* (PA3992) |  | pACCV-28 | pET28a(+) | Truncated aa 1-32 | E. coli BL21(DE3) Star |
| *PatB* (CJJ81176_0639) | *Campylobacter jejuni* 81-176 | pACAA5 | pET28a(+) | Truncated aa 1 - 31 | *E. coli* C43(DE3) |
| *PgdA* (CD630_15220) | *Clostridioides difficile* 630 | pBEDS-1 | pET28a(+) | Truncated aa 1 - 57 | E. coli BL21(DE3) |
| *NagZ* (BSU_01660) | *Bacillus subtilis 168* | pET-NagZ | pET28a(+) | Truncated 1 -18 | E. coli BL21(DE3) |
| *NamZ* (BSU_01650) |  | pET-NamZ | pET28a(+) | Truncated 1 -24 | E. coli BL21(DE3) |
| *LytD* (BSU_35780) |  | pET-LytDcat | pET28a(+) | Truncated aa 1-589 | E. coli BL21(DE3) pLysS |
| *AmiA* (AtlA amidase domain; SAUSA300_0955) | *Staphylococcus aureus* USA300 | pET-AmiA | pET-SUMO | Truncated aa 1-198 and aa 421-1256 | E. coli BL21(DE3) |

| **Table S3. Induction conditions used in the study** | | | |
| --- | --- | --- | --- |
| Protein | Temperature | Working [IPTG] | Duration |
| MltA | 37°C | 0.1 mM | 16 h |
| MltB |  | <https://pubs.acs.org/doi/10.1021/bi011833k> |  |
| MltD | 15°C | 0.1 mM | 16 h |
| MltF | 30°C | 1 uM | 16 h |
| MltF2 | 15°C | 0.1 mM | 16 h |
| RlpA | 15°C | 0.1 mM | 16 h |
| Slt70 | 15°C | 10 uM | 16 h |
| SltB1 |  | <https://pubs.acs.org/doi/10.1021/bi011833k> |  |
| SltB2 | 15°C | 0.4 mM | 16 h |
| SltB3 | 15°C | 1 uM | 16 h |
| MltG | 15°C | 0.2% arabinose | 16 h |
| PatB | 15°C | 1 mM | 16 h |
| PgdA | 37°C | 0.1 mM | 3 h |
| NamZ | 37°C | 1 mM | 3 h |
| NagZ | 37°C | 1 mM | 3 h |
| LytD | 16°C | 1 mM | 16 h |
| AmiA | 16°C | 1 mM | 16 h |

**Purification buffers and details:**

MltA, MltD, MltF2, RlpA

Lysis buffer (L1): 50 mM Tris pH 8.0, 300 mM NaCl, 10% glycerol, 0.05% Brij-35, 5 mM EDTA, 20 mM imidazole

Elution buffer (E1): L1 + 50-1000 mM imidazole

sMltF, SltB2, SltB3

Lysis buffer: 50 mM sodium phosphate pH 8.0, 300 mM NaCl, 10 % glycerol

Wash buffer 1: L1 + 0.1% Triton X-100 pH 7.0

Elution buffer 1: L1 + 0.1% Triton X-100 pH 5.0

Elution buffer 2: L1 + 0.1% Triton X-100 pH 4.5

MltG purification

Lysis buffer (L1): 50 mM Tris pH 8.0, 300 mM NaCl, 10% glycerol, 0.05% Brij-35, 5 mM EDTA, 20 mM imidazole

Following lysis, cell-free lysate was applied to a gravity column packed with Roche cOmplete His-Tag Purification Resin. Washes were performed with 50 mL L1 followed by 50 mL L1 containing no NaCl. After washing, 10 mL L1 was added, then an aliquot of SUMO protease. This was incubated at room temperature for 1 h with gentle nutation. Flowthrough was collected, concentrated and loaded onto a HiLoad^TM^ 16/600 Superdex^TM^ 200 pg column (Cytiva). Size-exclusion chromatography was performed in 10 mM sodium acetate pH 5.0, 10 mM MgCl_2_, 10% glycerol.

Slt70 purification

Lysis buffer: 50 mM sodium phosphate pH 8.0, 300 mM NaCl

Wash buffer 1: lysis buffer pH 7.0

Wash buffer 2: lysis buffer pH 6.5

Wash buffer 3: lysis buffer pH 6.0

Elution buffer 1: lysis buffer pH 5.0

Elution buffer 2: lysis buffer pH 4.5

Elution buffer 3: 250 mM sodium phosphate, 300 mM NaCl pH 8.0

IMAC was performed in a gravity column packed with TALON® Metal Affinity Resin (Takara Bio). For the final step, E2 was eluted into E3 to prevent protein precipitation. Dialysis was subsequently performed in 10 mM sodium acetate pH 4.0 , 10 mM MgCl_2_, 10 % glycerol.
